# Determination of risk factors for hepatitis B and C in male patients suffering from chronic hepatitis

**DOI:** 10.1186/1756-0500-2-212

**Published:** 2009-10-23

**Authors:** Huma Qureshi, Ambreen Arif, Kashif Riaz, Syed Ejaz Alam, Waquaruddin Ahmed, Syed Abdul Mujeeb

**Affiliations:** 1 Medilink and Orthopeadic and Medical Institute (OMI) Hospital Karachi, Karachi, Pakistan; 2 Pakistan Medical Research Council Research Centre, Jinnah Postgraduate Medical Centre, Karachi, Pakistan; 3 Blood Bank and Transfusion Services Jinnah Postgraduate Medical Centre, Karachi, Pakistan

## Abstract

**Background:**

Hepatitis B and C is common in Pakistan and various risk factors are attributable to its spread.

One thousand and fifty consecutive male cases suffering from chronic liver disease (327 HBV and 723 HCV) were selected from the OPD of public sector hospital and a private clinic dealing exclusively with the liver patients. To compare the results 723 age and gender matched controls were selected from the blood transfusion services of the public sector hospital. A standard questionnaire was filled for all patients and controls which included the information on possible risk factors.

**Findings:**

Family history of liver disease was significantly higher (43% and 34%) in HBV and HCV positive cases as compared to 5% in controls [odds ratio 15.6; 95% Confidence Interval CI: 10.1 -- 24.1, 10.9; 95% Confidence Interval CI: 7.3 -- 16.4] and same trend was seen for death due to liver disease in the family. Majority 74% hepatitis B positive cases had their shaves done at communal barbers but this practice was equally prevalent amongst controls (68%), thus negating it as a possible risk factor, but there is a significant risk with p < 0.05 associated with HCV in male that get their shave in barber. Very strong association of the disease was found with history of dental treatment (38% HCV 36% HBV and 21% controls) [Odd ratio 2.3; 95% CI: 1.8-3.0, Odd ratio 2.1; 95% CI: 1.5-2.8], surgery (23% HCV cases,14% HBV cases and 12% controls), history of blood transfusion was significantly higher in HCV (6%) as compared to controls (2.1%) [Odd ratio 2.9; 95% CI: 1.5-5.5]. History of taking injections for various ailments by the general practitioners (over 90% patients in both hepatitis B and C cases) was significantly higher as compared to 75% in controls [Odds ratio 3.8, 6.9; 95% CI: 2.4-6.1, 4.5-10.4] but hospitalization was not significant in HBV and HCV cases.

**Conclusion:**

Injections, surgery and dental treatment appear as major risk factors for the transmission of hepatitis B and C in the community. Massive health care awareness drives need to be done for both health care providers and the public to reduce this menace.

## Background

Hepatitis B and C are the major causes of chronic liver diseases in Pakistan. Using meta analysis of the previous 10 years data, it has been found that the prevalence of HBV in Pakistan is around 3-4% [1] and HCV is around 5-7% [2]. Some parts of the country show higher figures for both these viruses. Both viruses spread though blood and its products and body secretions. For the prevention of HBV a potent vaccine is available which has over 95% protection rates. No vaccine is available for HCV. In Pakistan the frequency of HCV appears to be increasing and the possible sources include frequent injections for minor ailments, shaving by barbers, dental treatments and blood transfusions along with surgeries. Improper sterilization of medical devices and reuse of syringes has been reported to be the major factor for this high increase in uncontrolled studies [3-6]. The present study was done to see if various risk factors mentioned above show a direct or indirect association with the HBV or HCV disease and to compare it with controls.

## Methods

One thousand and fifty consecutive male patients suffering from chronic hepatitis B or C liver disease and coming for treatment and follow-up to the Gastroenterology and Hepatology unit of Pakistan Medical Research Centre, Jinnah Postgraduate Medical Centre and a private hepatology clinic were selected for study. The diagnosis was confirmed by means of clinical data, deranged liver function tests and positive hepatic serology i.e. HBsAg or anti HCV tests. Seven hundred and twenty three age matched controls (family replacement blood donors) who were hepatitis B and C negative were selected from the blood bank of the Jinnah postgraduate medical centre. Informed consent was taken from patients and controls and ethical clearance was taken from the institutional review board. A questionnaire was filled for all cases and controls which contained the basic information about the individual like age, gender, past and family history of liver disease. To determine the possible sources of viral transmission history of dental treatment which included extraction, filling or scaling, surgery, blood transfusion, habit of taking intramuscular injections for minor ailments and shaving by communal barbers was also taken.

### Data management and analysis plan

Statistical Package for Social Sciences (SPSS) version 14.0 was used for data entry and analysis. The analysis was carried out two levels univariate and multivariate analysis. Mean and standard deviation (SD) were calculated for quantitative variable (age) and proportions for categorical variables. Univariate logistic regression analysis was performed to measure association of outcome with each independent variable. Odds ratios (OR) and 95% confidence intervals (CI) was calculated for each association. Associations among independent variables were assessed using appropriate test before performing multivariate analysis. Multiple logistic regression models were used to examine the association between the independent variables and the main outcomes variables, Hepatitis B and C virus while controlling for the effects of other covariates. P-value < 0.05 was considered as statistical significant

## Results

A total of 1050 patients suffering from chronic HBV or HCV related liver disease were included in the study. Of 1050 patients 327 were suffering from chronic hepatitis B (age range15 -- 60 years, mean ± S.D was 31.0 ± 9.4) and 723 from chronic hepatitis C (age range 16-65 years, mean ± S.D was 32.5 ± 8.4). Seven hundred and twenty three (723) age matched controls (age range 18-65 years, mean ± S.D was 32.0 ± 8.6) who were hepatitis B and C negative were selected from the blood bank of the Jinnah Postgraduate Medical Centre. Forty three HBV positive cases and 34% HCV positive cases had family history of liver diseases which was significantly higher when compared to 5% in controls (P < 0.001). Death in the family due to liver disease was seen in 5.2% cases having HBV and 4.3% in HCV, which was not statistically significant when compared with 3% seen in controls (Table 1, 2).

**Table 1:** Risk factors associated with Hepatitis B in Males

**Risk factors**		**HBV** **(n = 327)**		**Controls** **(n = 723)**		**Odds Ratio (OR)** **95% Confidence Interval (CI)**	
		**No**	**%**	**No**	**%**		**P-value**
Any family member suffering from hepatitis B or C	Yes	140	42.8	33	4.6	15.6 (10.1-24.1)	0.001
	No	187	57.2	690	95.4		
Any family member died of liver disease	Yes	17	5.2	22	3.0	1.7 (0.8-3.4)	0.124
	No	310	94.8	701	97.0		
Where do you get your shave	No	25	7.6	72	10.0	Reference	
	Home	59	18.0	159	22.0	1.1 (0.6-1.9)	0.919
	Barber	243	74.3	492	68.0	1.4 (1.0-2.3)	0.184
Dental extract/filling/scaling	Yes	117	35.8	151	20.9	2.1 (1.5-2.8)	0.001
	No	210	64.2	572	79.1		
Surgery	Yes	47	14.4	82	11.3	1.3 (0.8-1.9)	0.199
	No	280	85.6	641	88.7		
Blood Transfusion	Yes	13	4.0	15	2.1	1.9 (0.8-4.4)	0.118
	No	314	96.0	708	97.9		
History of IV/IM injection	Yes	302	92.4	548	75.8	3.8 (2.4-6.1)	0.001
	No	25	7.6	175	24.2		
Number of IV/IM injections	None	25	7.6	175	24.2	Reference	
	< 5/year	136	41.6	383	53.0	2.5 (1.5-4.0)	0.001
	≥ 5/year	166	50.8	165	22.8	7.0 (4.3-11.6)	0.001
Type of syringe	New	16	4.9	178	24.6	8.6 (4.9-15.2)	0.001
	Re-used	286	87.5	370	51.2		
History of Hospitalization	Yes	38	11.6	98	13.6	0.8 (0.5-1.2)	0.444
	No	289	88.4	625	86.4		

Statistically significant p < 0.05

**Table 2:** Risk factors associated with Hepatitis C in Males

**Risk factors**		**HCV** **(n = 723)**		**Controls** **(n = 723)**		**Odds Ratio (OR)** **95% Confidence Interval (CI)**	**P-value**
		**No**	**%**	**No**	**%**		
Any family member suffering from hepatitis B or C	Yes	249	34.4	33	4.6	10.9 (7.3-16.4)	0.001
	No	474	65.6	690	95.4		
Any family member died of liver disease	Yes	31	4.3	22	3.0	1.4 (0.7-2.5)	0.263
	No	692	95.7	701	97.0		
Where do you get your shave	No	52	7.2	72	10.0	Reference	
	Home	124	17.2	159	22.0	1.1 (0.6-1.6)	0.807
	Barber	547	75.7	492	68.0	1.5 (1.0-2.4)	0.031
Dental extract/filling/scaling	Yes	277	38.3	151	20.9	2.3 (1.8-3.0)	0.001
	No	446	61.7	572	79.1		
Surgery	Yes	164	22.7	82	11.3	2.3 (1.7-3.1)	0.001
	No	559	77.3	641	88.7		
Blood Transfusion.	Yes	42	5.8	15	2.1	2.9 (1.5-5.5)	0.001
	No	681	94.2	708	97.9		
History of IV/IM injection	Yes	691	95.6	548	75.8	6.9 (4.5-10.4)	0.001
	No	32	4.4	175	24.2		
Number IV/IM injection/year	None	32	4.4	175	24.2	Reference	
	<5/year	239	33.1	383	53.0	3.4 (2.2-5.2)	0.001
	≥ 5/year	452	62.5	165	22.8	14.9 (9.7-23.2)	0.001
Type of syringe	New	17	2.4	178	24.6	19.0 (11.1-33.0)	0.001
	Re-used	674	93.2	370	51.2		
History of Hospitalization	Yes	106	14.7	98	13.6	1.1 (0.8-1.4)	0.596
	No	617	85.3	625	86.4		

Of the total males only 18% in each group (HBV/HCV) had their shave done at home while the rest 74% and 76% had it done exclusively by barbers or both (home and barbers). There was a significant risk (p < 0.05) of contracting HCV infection in males that were getting their shave from communal barbers. History of dental extraction/filling/scaling was significantly higher (38%) in HCV and (36%) HBV infected cases (P < 0.001), when compared with controls (21%) Table 1, 2.

Past history of any kind of invasive surgery was present in 23% of HCV and 15% of HBV positive cases when compared with (11%) controls (p < 0.001). History of blood transfusion was 6% in HCV and 4% in HBV positive cases and these figures were significantly higher as compared to 2.1% in controls (p < 0.001).

History of therapeutic injection use for various ailments including intravenous drips was present in over 90% patients suffering from either hepatitis B or C as compared to 76% seen in controls, the difference was statistically significant (P < 0.001), and in majority of the cases injections were given by general practitioners by their own syringe. Only 2% of HCV and 5% of HBV positive cases said that they took their own syringe for injections (Table 1, 2). History of past hospitalization was present in 15% cases suffering from HCV and 12% HBV as compared to 14% seen in controls (Table 1, 2).

Multivariate logistic regression analysis showed 4 variables i.e. family members suffering from liver disease, dental treatments, blood transfusion and number of therapeutic injections taken by each person per annum, as significant risk factors for acquiring hepatitis B or c infection (table 3, 4)

**Table 3:** Independent risk factors associated with Hepatitis B in males using multivariate logistic regression

**New**				
**Risk Factor**		**Adjusted Odds Ratio**	**95% Confidence Interval**	**P-value**

Any family member suffering from hepatitis B & C	Yes	14.5	9.5-22.1	0.001
	No	Reference		
Dental extract/filling/scaling	Yes	1.9	1.4-2.7	0.001
	No	Reference		
Blood transfusion	Yes	2.9	1.2-7.3	0.017
	No	Reference		
History of IV/IM injection/year	None	Reference		
	< 5/year	2.1	1.3-3.4	0.003
	≥/year	5.8	3.5-9.6	0.001

**Table 4:** Independent risk factors associated with Hepatitis C in males using multivariate logistic regression

**New**				
**Risk Factor**		**Adjusted Odds Ratio**	**95% Confidence Interval**	**P-value**

Any family member suffering from hepatitis B & C	Yes	10.1	6.8-15.1	0.001
	No	Reference		
Dental extract/filling/scaling	Yes	2.2	1.6-2.8	0.001
	No	Reference		
Blood transfusion	Yes	4.6	2.1-9.7	0.001
	No	Reference		
History of IV/IM injection/year	None	Reference		
	< 5/year	3.0	1.9-4.7	0.001
	≥/year	12.9	8.3-20.1	0.001

## Comments

In the present study a total of 1050 male patients with chronic HBV or HCV and 723 ages matched controls were studied. Only male patients were selected for the study because it is a male dominated society where shaving is a pure male ordeal. Blood donors were taken as controls because they represent the same catchments as of patients and generally are similar in age as patients. Of the patients 327 were suffering from chronic hepatitis "B" and 723 from hepatitis "C" giving a 2 times more occurrence of HCV than HBV in our population.

In Pakistan HBV exposure in neonates and children occurs through maternal transmission or as horizontal transmission from adults [7-9]. The exposure leads to persistence of infection which later in adulthood is seen either as healthy carriers or if the virus is active then it shows as chronic liver disease. Exposure in adults leads to either acute infection with recovery or no disease if the individual is immune to the virus [10]. Less than 5% adults become carriers following acute infection with HBV; so HBV infection in Pakistan is a problem of neonates and children. For HCV infection there is no immunity and once exposed about 60-70% will develop the disease. This infection is rare in children as the common sources of infection like shaving, operations, blood transfusions and injections are less frequent in childhood. In the present study the HBV to HCV ratio was 2:1 in those who were less than 35 years of age but this ratio reversed to 1:2 as the age advanced to 35 years and above. The possible reasons for high occurrence of HBV in younger age are due to at birth or neonatal exposure. As the age advances these cases either seroconvert or suffer and die leading to reduction in number of positive cases in advancing ages. HCV exposure usually starts later in life (adolescent life) due to exposure to above mentioned procedures, therefore we see more cases of HCV as the age advances. History of chronic liver disease in the family was seen in 43% HBV cases and this includes maternal (vertical) and interfamilial (horizontal) transmission of HBV and is in agreement with the previous reports of interfamilial transmission [11]. HCV (34%) infection runs in families in Pakistan and this is probably due to transmission of the disease from a common source i.e. barber, general practitioner using un-sterilized syringes and invasive medical devices.

Shaving by barbers, use of injections for common ailments, blood transfusion, surgery and dental treatments are thought to be the potential sources of transmission of HCV in Pakistan. In the present study only 18% cases were either shaving at home or had beards and were therefore not shaving, rest of the 75% were shaving at barbers. These figures were similar when compared with the general population (controls) thus negating communal shave as the source of transmission of the disease.

Intramuscular injections for common ailments and intravenous injections as drips for various reasons are used by majority of the people. Over 90% cases gave history of taking injections and that also by the syringe of the general practitioners and only 5% HBV and 2% HCV cases took their own syringe. Over 30% cases in both the groups gave history of taking 5-10 injections per year, while 15% and 24% of HBV and HCV cases were taking over 20 injections/year, which was quite high when compared with the controls (2%). The reuse of disposable syringe is still being practiced by most health care providers all over Pakistan.

History of surgery of any kind was twice more common in HCV positive cases vs. HBV cases and when compared with the controls this was very high (p = 0.001). Of the various kinds of surgeries; gall bladder surgery was the commonest. Indigestion is a major presenting feature of HCV infected cases and often an ultrasound is done to determine the cause. Once gall stones are found surgery is done, so more gall bladder surgery in HCV cases might be related to symptom complex rather than the organ itself. Apart from surgeries which were done more in HCV cases the fatigue and weakness seen in HCV cases might have been a provoking factor for taking a transfusion. Weakness as a good reason to take a transfusion was also shown in a study done by Khan in 2000 [12].

Though HBV vaccination is available in Pakistan for over 2 decades but still vaccination in adults is lower than expected. Only 6.7% controls gave history of being vaccinated against hepatitis B as against 16% HCV infected cases. Higher vaccination figures in HCV cases might be due to advice of the consultant to give prophylaxis before starting interferon therapy.

Taking preventive therapy as the best option to control disease spread, it is suggested that more emphasis should be laid down on HBV vaccination of all infants and children. For HCV control public education and awareness needs to be drastically scaled [13] and public made aware that equally effective oral medications are available when required and injections are no better than oral medications. Shaving by barbers though is not standing out as the possible risk factor but this practice still needs to be discouraged and barbers need to be educated on simple and easy ways for sterilization of instruments. Sterilization of invasive medical devices like endoscopes (respiratory, gut, gynecology, ENT, bones, laparoscopes etc), surgical instruments, dental instruments etc need to be done according to the standard SOPs. More potent but yet ignored sources of transmission include thermometers in GP clinics, tongue depressors, suction catheters and drain tubes/bags etc which are used from patient to patient without changing or cleaning. It is suggested that as a start the government should make it mandatory that all injections should available as single dose and multidose injection vials should be discouraged; moreover each vial should have a complimentary syringe attached to it. Later the GP clinics, hospitals and all places where health care is being provided should be provided with huge quantities of disposable syringes at a nominal or no cost basis for at least 3-5 years so that the desire to reuse a syringe can be overcome. Meanwhile increasing awareness shall improve the situation in the next few years.

## List of abbreviations

HBV: Hepatitis B Virus; HCV: Hepatitis C Virus; HBsAg: Hepatitis B Surface Antigen; Anti HCV: Antibody to Hepatitis C Virus; GP: General Practitioner.

## Competing interests

The authors declare that they have no competing interests.

## Authors' contributions

HQ conceived the study, participated in its design and coordination by sharing patient data from two private clinics and wrote the manuscript. AA participated in data collection and compilation from the public sector hospital. KR participated in data collection from private clinics. SEA participated in the design of the study and performed the statistical analysis. WA participated in sharing patient data from public sector hospital. SAM participated in sharing data of controls from Blood Bank of public sector hospital. All authors read and approved the final manuscript
